# Common Core Genes Play Vital Roles in Gastric Cancer With Different Stages

**DOI:** 10.3389/fgene.2022.881948

**Published:** 2022-07-22

**Authors:** Zhiyuan Yu, Chen Liang, Huaiyu Tu, Shuzhong Qiu, Xiaoyu Dong, Yonghui Zhang, Chao Ma, Peiyu Li

**Affiliations:** ^1^ School of Medicine, Nankai University, Tianjin, China; ^2^ Department of General Surgery, The First Medical Center, Chinese PLA General Hospital, Beijing, China; ^3^ First Department of Liver Disease / Beijing Municipal Key Laboratory of Liver Failure and Artificial Liver Treatment Research, Beijing You’an Hospital, Capital Medical University, Beijing, China

**Keywords:** gastric cancer, microarray datasets, differentially expressed genes, metabolism, meta-analysis

## Abstract

**Background** **:** Owing to complex molecular mechanisms in gastric cancer (GC) oncogenesis and progression, existing biomarkers and therapeutic targets could not significantly improve diagnosis and prognosis. This study aims to identify the key genes and signaling pathways related to GC oncogenesis and progression using bioinformatics and meta-analysis methods.

**Methods:** Eligible microarray datasets were downloaded and integrated using the meta-analysis method. According to the tumor stage, GC gene chips were classified into three groups. Thereafter, the three groups’ differentially expressed genes (DEGs) were identified by comparing the gene data of the tumor groups with those of matched normal specimens. Enrichment analyses were conducted based on common DEGs among the three groups. Then protein–protein interaction (PPI) networks were constructed to identify relevant hub genes and subnetworks. The effects of significant DEGs and hub genes were verified and explored in other datasets. In addition, the analysis of mutated genes was also conducted using gene data from The Cancer Genome Atlas database.

**Results:** After integration of six microarray datasets, 1,229 common DEGs consisting of 1,065 upregulated and 164 downregulated genes were identified. Alpha-2 collagen type I (COL1A2), tissue inhibitor matrix metalloproteinase 1 (TIMP1), thymus cell antigen 1 (THY1), and biglycan (BGN) were selected as significant DEGs throughout GC development. The low expression of ghrelin (GHRL) is associated with a high lymph node ratio (LNR) and poor survival outcomes. Thereafter, we constructed a PPI network of all identified DEGs and gained 39 subnetworks and the top 20 hub genes. Enrichment analyses were performed for common DEGs, the most related subnetwork, and the top 20 hub genes. We also selected 61 metabolic DEGs to construct PPI networks and acquired the relevant hub genes. Centrosomal protein 55 (CEP55) and POLR1A were identified as hub genes associated with survival outcomes.

**Conclusion:** The DEGs, hub genes, and enrichment analysis for GC with different stages were comprehensively investigated, which contribute to exploring the new biomarkers and therapeutic targets.

## Introduction

Gastric cancer (GC) is the third leading cause of cancer deaths and the fifth most frequently diagnosed cancer worldwide, with over 1 million new cases and 700,000 deaths each year (Bray et al., 2018). Most GC patients are already in the advanced stage at the initial visit, and these patients have a poor prognosis and even have to undergo radical surgery (Irino et al., 2021; Manzanedo et al., 2021; Stocker et al., 2021). In the past decades, gene sequencing and molecular targeted therapy have been increasingly widely used in clinical practice. Therefore, it is of great clinical value to explore the core genes and molecular mechanisms in pathogenesis for the diagnosis and treatment of GC.

In an analysis of key circulating exosomal miRNAs, four key miRNAs (hsa-miR-130b-3p, hsa-miR-151a-3p, hsa-miR-15b-3p, and hsa-miR-1246) and the interaction network or enrichments based on their targets (TAOK1, CMTM6, SCN3A, WASF3, IGF1, CNOT7, GABRG1, and PRKD1) may help understand the molecular mechanisms in the GC development (Qian et al., 2021). The study by Mou et al. (2015)demonstrated that four SNP loci (rs2279115, rs804270, rs909253, and rs3765524) showed a potential association with GC risk. In addition, many targets and markers have been applied to the diagnosis and treatment of GC in basic experiments and achieved preliminary results. HER2 monoclonal antibody conjugated RNase-A-associated CdTe quantum dots (Ruan et al., 2012) and BRCAA1 monoclonal antibody conjugated fluorescent magnetic nanoparticles (Wang et al., 2011) both exhibited great potential in applications such as *in situ* GC targeted imaging and selective therapy of GC. The study by Zhang et al. (2015)showed that miR-19b-3p and miR-16-5p were biomarkers that own great potential in applications such as early screening and progression evaluation of GC. Furthermore, the microarray-based prewarning system, which could be applied in the early detection of GC, was developed by Cui et al. (2005).

At present, many bioinformatic studies about GC have been published, but the differentially expressed genes (DEGs) and signaling pathways revealed by different studies were not consistent. The study by Yang and Gong (2021) showed that OLFM4, IGF2BP3, CLDN1, and MMP1 were the most significantly upregulated DEGs, which significantly enriched in negative regulation of growth, fatty acid binding, and cellular response to zinc ions. In a bioinformatics analysis conducted by Xu et al. (2021), the expressions of ITGB1 and alpha-2 collagen type I (COL1A2) were significantly increased in GC tissues, and 63 characteristic DEGs were mainly involved in regulating extracellular matrix (ECM)–receptor interactions and the PI3K-Akt signaling pathway. Xu et al. (2020) also found that SLC1A3 promotes GC progression via the PI3K/AKT signaling pathway.

Most published experimental and bioinformatic studies included GC specimens with unclear tumor stages, making it impossible to accurately analyze the DEGs and signaling pathways throughout GC development (Yang et al., 2021; Xu et al., 2021). In this study, therefore, we retrieved three microarray datasets containing gene data with definite GC stages and then divided them into the early stage (ES) group and the late stage (LS) group. The two groups’ DEGs were obtained by comparing GC tissues of the ES and LS groups with adjacent noncancerous gastric tissues. Another three microarray datasets containing gene data with indefinite GC stages were also collected, and DEGs in GC were identified relative to normal tissues. Gene Ontology (GO) and Kyoto Encyclopedia of Genes and Genomes (KEGG) pathway enrichment analyses were conducted on DAVID (https://david.ncifcrf.gov/) and Sangerbox 3.0 (http://vip.sangerbox.com/home.html) using common DEGs from the three groups. Thereafter, protein–protein interaction (PPI) network was constructed using the STRING online tool (https://cn.string-db.org/) and Cytoscape software (Shannon et al., 2003). The effects of significant DEGs and hub genes were verified and explored in other datasets. The related gene expression data and clinical information were also obtained from The Cancer Genome Atlas (TCGA) database (https://portal.gdc.cancer.gov/), which were used to carry out overall survival analysis and somatic mutation analysis. Besides this, hub genes associated with metabolic KEGG pathways were also identified. Through this analysis, we identified the key genes and signaling pathways related to GC, aiming to provide the experimental basis and important insight of new biomarkers and prognostic markers.

## Materials and Methods

### Inclusion and Integration of Microarray Datasets

Eligible microarray datasets were downloaded from the Gene Expression Omnibus (GEO) database (http://www.ncbi.nlm.nih.gov/geo/). Datasets in accordance with the following criteria were included and considered for subsequent analysis: upload data were between 2010 to 2021; contained the gene data on GC tissues and adjacent normal tissues and at least three samples per group; tissue samples used were from humans; and detail information on technology and platform were obtainable.

When the staging of GC specimens was definite, gene chips were divided into the ES group and LS group based on tumor stages. The ES group and LS group respectively incorporated stage I–II and stage III–IV GC’s microarray data, which were staged according to standards recommended by the American Joint Committee on Cancer. GC gene chips with indefinite tumor stages were classified as the mixed stage (MS) group, incorporating stage I–IV GC’s microarray data. DEGs were obtained by comparing the microarray data of GC with those of adjacent noncancerous gastric tissues. Thereafter, meta-analysis was performed to integrate microarray data for the above each group, and the three groups’ DEGs would be obtained. DEGs from the three groups were intersected to identify common DEGs. In addition, DEGs were ranked according to adjusted *p*-value (adj.P.Val) from small to large, and the most significant DEGs were also gained by intersecting the top 100 significant DEGs from the three groups. The effects of above significant DEGs were verified and explored in other GEO datasets (GSE103236, GSE51725, and GSE17187) and TCGA database.

### Enrichment Analysis of Gene Ontology Terms and Kyoto Encyclopedia of Genes and Genomes Pathways

**FIGURE 1:**
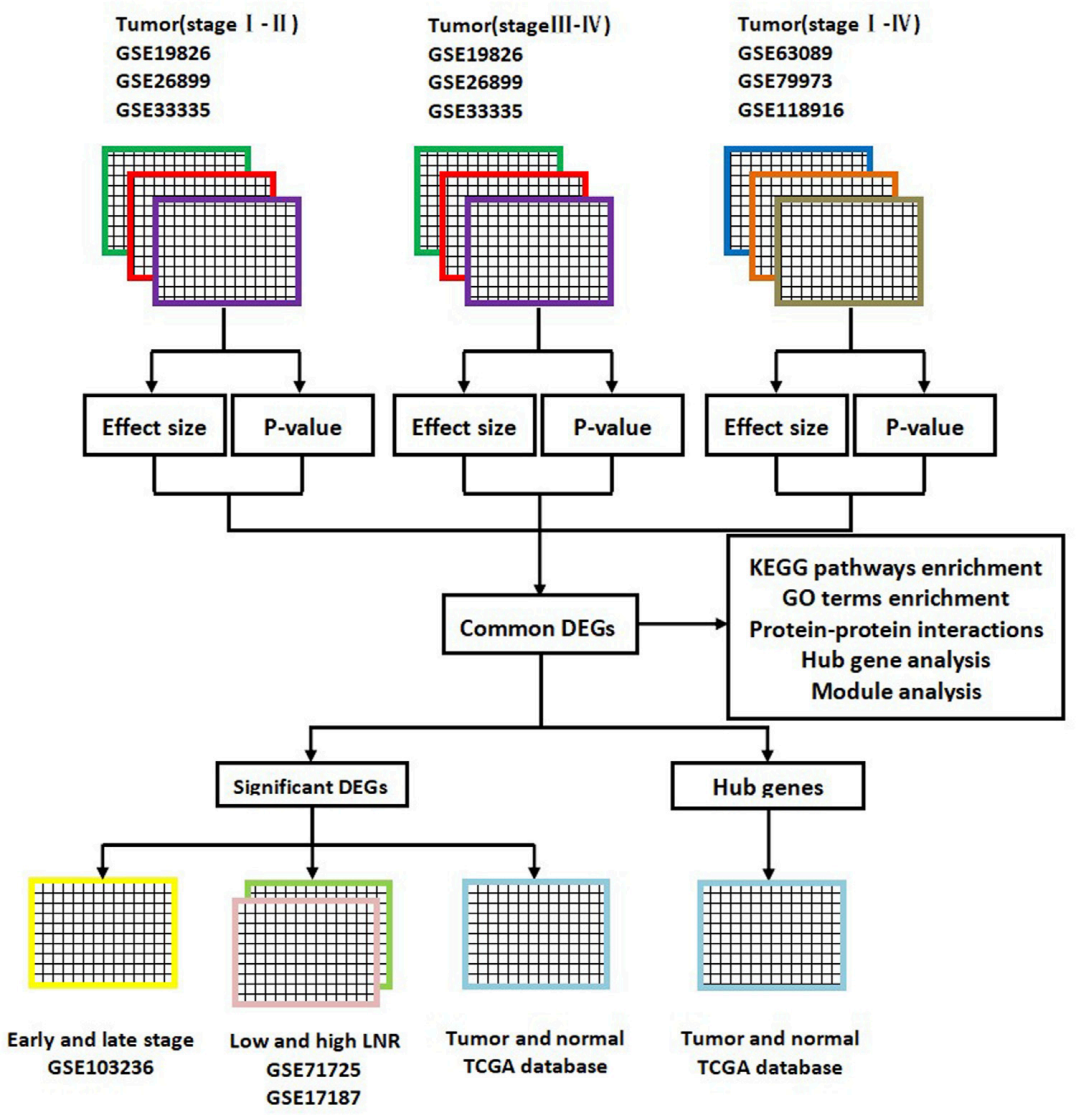
Flow diagram of this bioinformatics and meta-analysis. DEGs, differentially expressed genes; KEGG, Kyoto Encyclopedia of Genes; GO, gene ontology; LNR, lymph node ratio; TCGA, The Cancer Genome Atlas.

Common DEGs were divided into the upregulated group and the downregulated group. Next, GO terms and KEGG pathways enrichment analyses of upregulated DEGs and downregulated DEGs were respectively performed using the DAVID online tool. GO terms consisted of the following items: biological process (BP), molecular function (MF), and cellular component (CC). KEGG pathway analysis was designed to identify significantly enriched pathways of molecular interactions and reactions. The flow diagram of this bioinformatics and meta-analysis is shown in Figure 1.

### Establishment and Analysis of Protein–Protein Interaction Network

The PPI network of DEGs was constructed based on the STRING database, with a confidence score set as 0.9 (highest confidence). Then, PPI network files were imported to Cytoscape v3.8 software, in which we constructed the subnetwork using the MCODE plug-in and calculated the top 20 genes based on the Multiscale Curvature Classification (MCC) algorithm. The DAVID, Metascape (https://metascape.org/), and Sangerbox 3.0 online tools were used for enrichment analysis and visualization of hub genes and the first subnetwork. The effects of hub genes were explored and proved in another three GEO microarray datasets (GSE103236, GSE51725, and GSE17187) and TCGA database. The DEGs converged on KEGG pathways associated with metabolism and were also inputted into the STRING database and Cytoscape software to obtain relevant PPI networks and hub genes.

### Statistical Analysis

Integrative meta-analysis of GEO data (ImaGEO) (https://imageo.genyo.es/), an online tool, was adopted to integrate microarray data (Toro-Domínguez et al., 2019). What is more, the fixed effect model of the effect size method and Fisher’s *p*-value method were both applied to perform a meta-analysis for each group. The adj.P.Val < 0.05 and |Log2 fold change (logFC)| > 1 were set as the parameters to identify DEGs. The Wilcoxon test was employed to compare the statistical differences between the two groups, while the Kruskal–Wallis (KS) test was adopted as a statistical method to compare multiple groups. And beyond that, all cut-off *p*-values were set as *p* < 0.05 in this analysis.

## Results

### Microarray Dataset Integration and Differentially Expressed Gene Identification

First, the microarray datasets containing GSE19826, GSE26899, GSE33335, GSE63089, GSE79973, and GSE118916 were acquired for further analyses. Inadaptable gene chips were removed from selected microarray datasets. After selection, the expression data of 42 GC tissues from GSE19826, GSE26899, and GSE33335 were classified as the ES group, while the expression data of another 53 GC tissues from the above same microarray datasets were classified as the LS group. In addition, GSE63089, GSE79973, and GSE118916 respectively included the gene data of 45, 10, and 15 GC tissues and were classified as the MS group. The characteristics of selected GEO microarray datasets are shown in Table 1.

**TABLE 1:** Characteristics of included GEO datasets.

Datasets	Platforms	Region	Sample size (Tumors) ES Group LS Group MS group			Sample Size (Controls)	Submission Date (year)
GSE19826	GPL570	China	6	6	—	12	2010
GSE26899	GPL6947	United States	23	36	—	12	2011
GSE33335	GPL5175	China	13	11	—	24	2011
GSE63089	GPL5175	China	—	—	45	45	2014
GSE79973	GPL570	China	—	—	10	10	2016
GSE118916	GPL15207	China	—	—	15	15	2018

GEO, Gene Expression Omnibus; GSE, GEO datasets; GPL, GEO platforms; ES, early stage; LS, late stage; MS, mixed stage.

**FIGURE 2:**
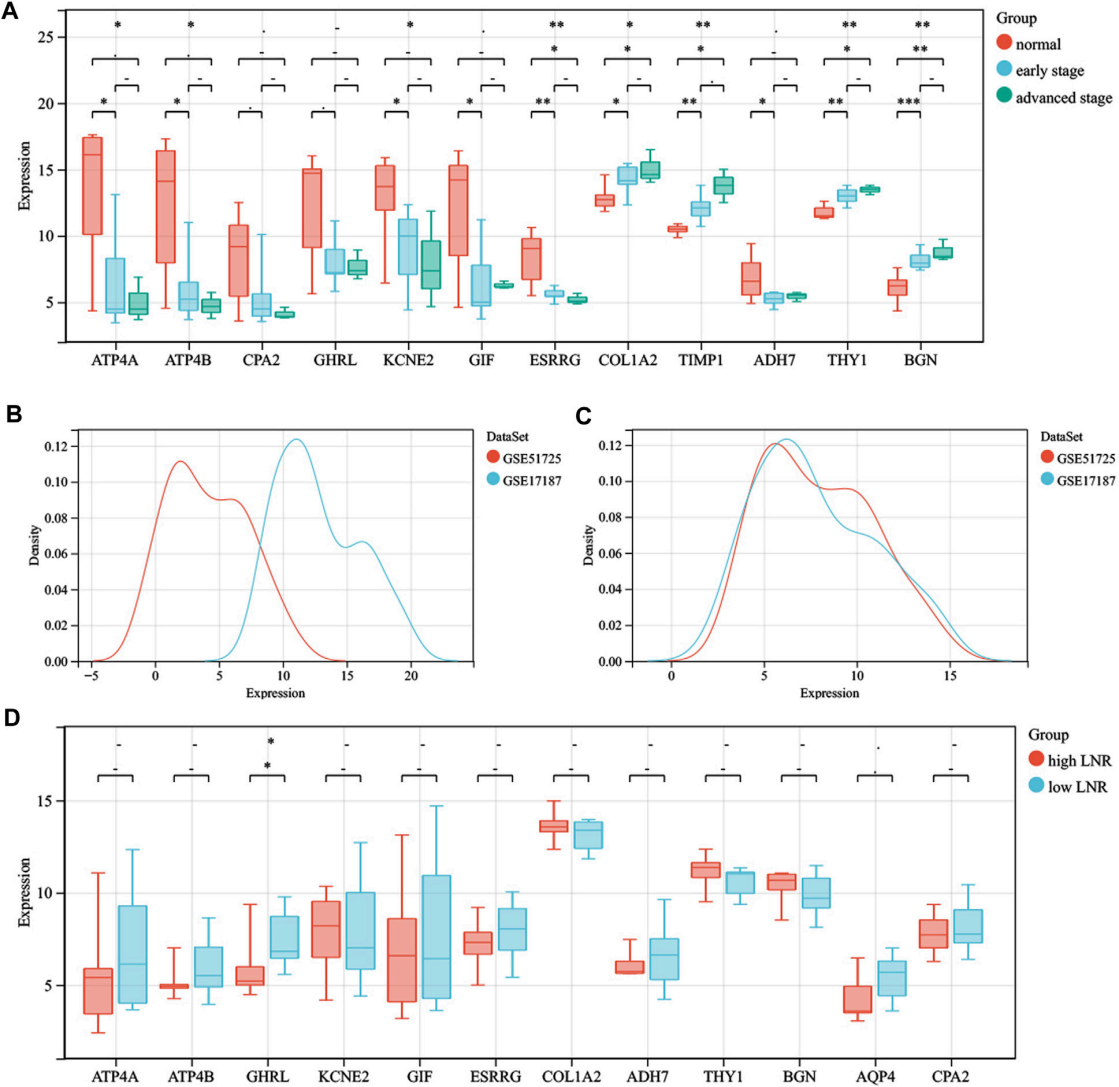
The roles that significant DEGs play in the oncogenesis, progression, and lymph node metastasis. **(A)** comparison of the expression levels of ATP4A, ATP4B, CPA2, GHRL, KCNE2, GIF, ESRRG, COL1A2, TIMP1, ADH7, THY1, and BGN between paracancer tissues, GC tissues with early stage and GC tissues with late stage. **(B)** gene data before normalization. **(C)** gene data after normalization. **(D)** comparison of the expression levels of ATP4A, ATP4B, GHRL, KCNE2, GIF, ESRRG, COL1A2, ADH7, THY1, BGN, AQP4, and CPA2 between GC tissues with high LNR and GC tissues with low LNR. The statistical methods adopted for A and D are the Kruskal–Wallis test and Wilcoxon test, respectively. ^**-**^, *p* > 0.05; *, *p* < 0.05; **, *p* < 0.01. DEGs, differentially expressed genes; GC, gastric cancer; LNR, lymph node ratio.

**FIGURE 3:**
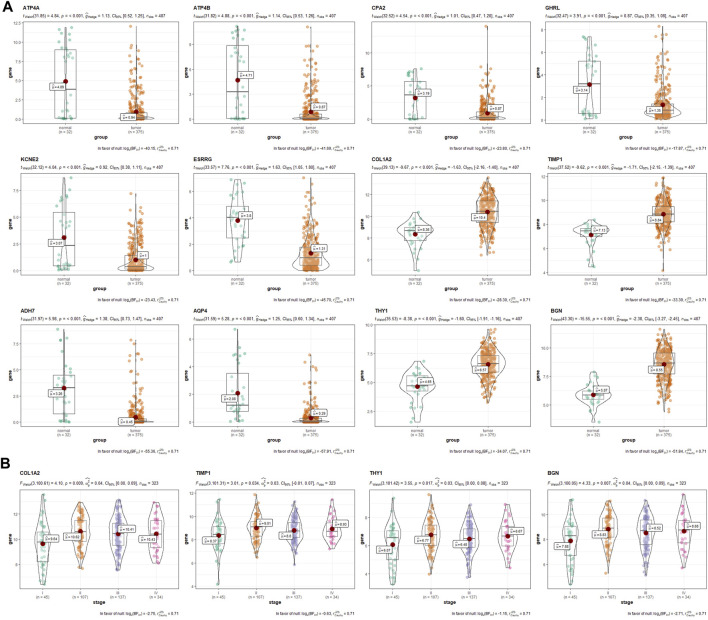
The verification and exploration of significant DEGs in TCGA database. **(A)** comparison of the expression levels of ATP4A, ATP4B, CPA2, GHRL, KCNE2, ESRRG, COL1A2, TIMP1, ADH7, AQP4, THY1, and BGN between GC and paracancer tissues from TCGA database. **(B)** the association between the expression levels of COL1A2, TIMP1, THY1, and BGN with tumor stage. DEGs, differentially expressed genes; TCGA, The Cancer Genome Atlas; GC, gastric cancer.

After intersecting the DEGs obtained using two different meta-analysis methods, 4,125, 3,699, and 3,531 DEGs were gained from the ES, LS, and MS groups, respectively. Then we intersected the above DEGs and identified 1,229 common DEGs, which consisted of 1,065 upregulated genes and 164 downregulated genes. In addition, ATP4A, ATP4B, CPA2, ghrelin (GHRL), KCNE2, GIF, ESRRG, COL1A2, tissue inhibitor matrix metalloproteinase 1 (TIMP1), ADH7, AQP4, thymus cell antigen 1 (THY1), and biglycan (BGN) were identified by intersecting the top 100 significant DEGs from the three groups. GSE103236, GSE51725, and GSE17187 were used to verify the effects of the above 12 DEGs (except AQP4) in tumorigenesis and development. By analyzing the gene data of GSE103236, we found that the expression levels of ESRRG, COL1A2, TIMP1, THY1, and BGN in GC tissues with ES and GC tissues with advanced stage were both significantly different than those in paracancer tissues (Figure 2A). GSE51725 and GSE17187 based on the same GEO platform (GPL570) were normalized, and the batch effect was removed. Compared with the expression data before normalizing (Figure 2B), those after removing the batch effect were found to be more consistent (Figure 2C). In the analysis of integrated gene data, a noticeable correlation was found between the expression level of GHRL and the LNR of the tumor (Figure 2D). Moreover, analysis based on TCGA database showed that the expression levels of 12 significant DEGs (except GIF) were significantly different between GC tissues and adjacent normal gastric tissues (Figure 3A), and statistical correlation could also be found between the expression levels of COL1A2, TIMP1, THY1, and BGN and tumor stage (Figure 3B).

### Enrichment Analysis Based on Common Differentially Expressed Genes

**FIGURE 4:**
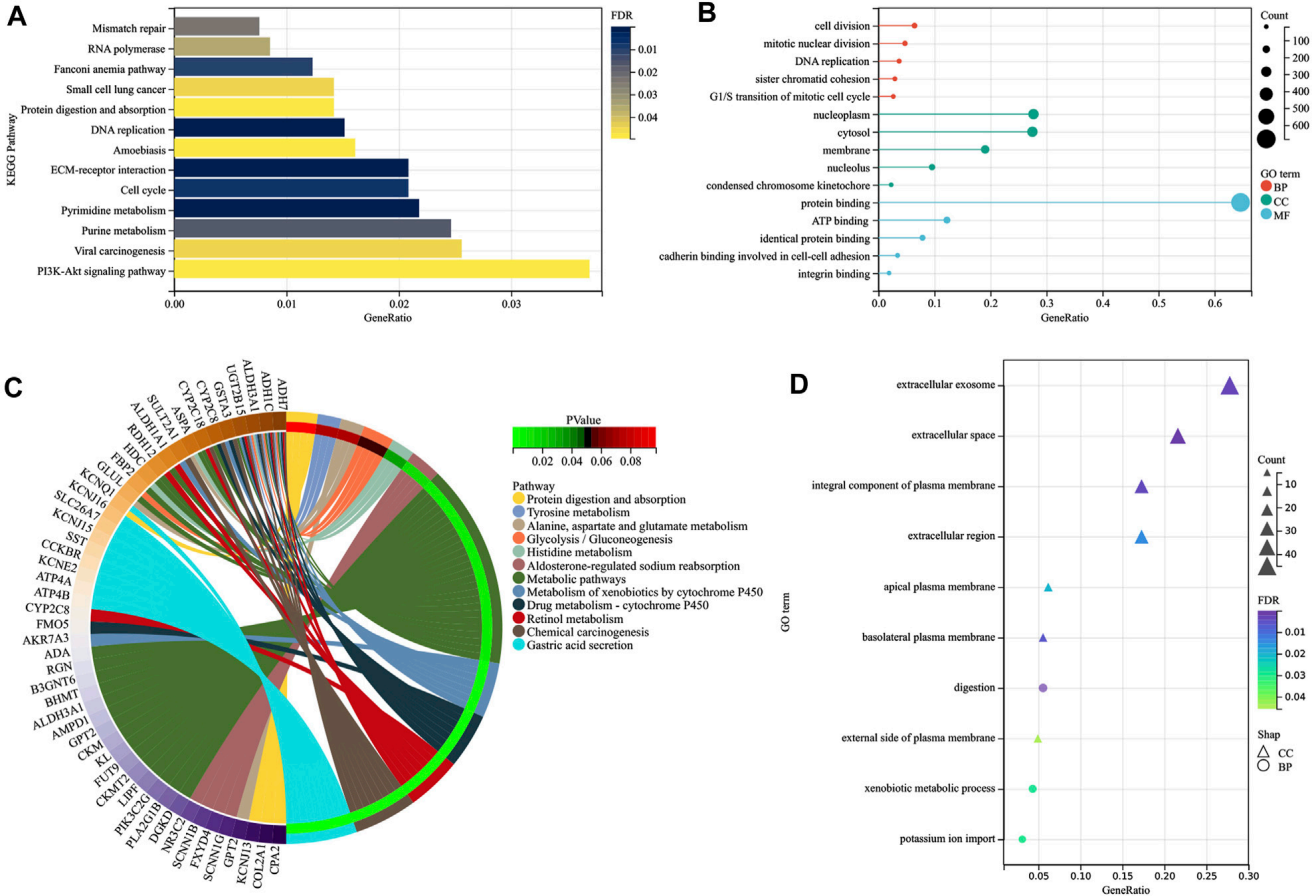
Visualization of the enrichment results. Distribution of the upregulated DEGs in GC for different **(A)** KEGG pathways and **(B)** GO-enriched functions, and distribution of the down -regulated DEGs in GC for different **(C)** KEGG pathways and **(D)** GO-enriched functions. DEGs, differentially expressed genes; GC, gastric cancer; KEGG, Kyoto Encyclopedia of Genes and Genomes; GO, Gene ontology; BP, biological process; CC, cellular component; MF, molecular function.

Enrichment analyses of KEGG pathways and GO terms were both performed for upregulated and downregulated DEGs using the DAVID online tool. Regarding upregulated DEGs, KEGG pathway analysis showed that DEGs were mainly enriched in DNA replication, ECM–receptor interaction, pyrimidine metabolism, cell cycle, and purine metabolism (Figure 4A), while DEGs were mainly enriched in cell division, DNA replication, mitotic nuclear division, sister chromatid cohesion, and G1/S transition of the mitotic cell cycle in GO terms analysis (Figure 4B). As for downregulated DEGs, gastric acid secretion, chemical carcinogenesis, retinol metabolism, drug metabolism–cytochrome P450 and metabolism of xenobiotics by cytochrome P450 were KEGG pathways that play important roles (Figure 4C), while GO terms analysis showed that DEGs were mainly enriched in digestion, the xenobiotic metabolic process, potassium ion import, and gastric acid secretion (Figure 4D). It was also found that most KEGG pathways of downregulated DEGs were related to metabolism, including retinol metabolism, drug metabolism–cytochrome P450, metabolism of xenobiotics by cytochrome P450, metabolic pathways, glycolysis/gluconeogenesis, histidine, alanine, aspartate, glutamate, and tyrosine metabolism. In the analysis of upregulated DEGs, pyrimidine metabolism and purine metabolism were KEGG pathways associated with metabolism. In the end, 31 upregulated DEGs and 30 downregulated DEGs pooled in metabolism-related KEGG pathways were selected for further analysis.

### Protein–Protein Interaction Network Construction and Hub Gene Identification

**FIGURE 5:**
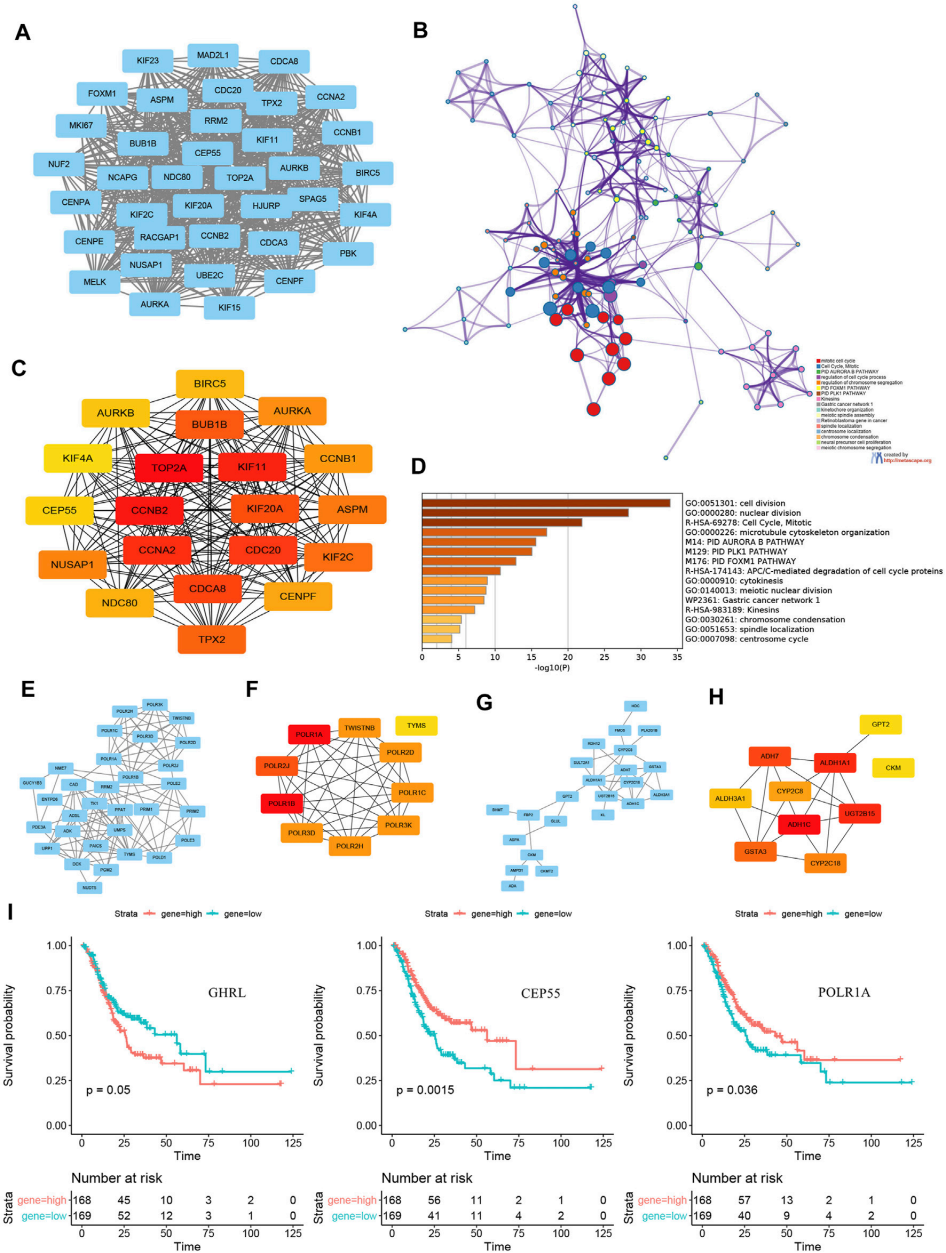
PPI subnetwork construction and hub genes identification. **(A)**the first subnetwork of PPI using MCODE plug-in of Cytoscape. **(B)** enrichment analysis and visualization of first cluster using Metascape online tool. **(C)** top 20 hub genes of all common DEGs identified by an MCC algorithm. **(D)** enrichment analysis and visualization of top 20 hub genes using the Metascape online tool. PPI networks and relevant hub genes of **(E–F)** 31 upregulated metabolic DEGs and **(G–H)** 30 downregulated metabolic DEGs. **(I)** survival curves of GHRL, CEP55, and POLR1A expression. PPI, protein–protein interaction; DEGs, differentially expressed genes; MCC, Multiscale Curvature Classification.

The PPI network of 1,229 common DEGs, which involved 656 nodes (DEGs) and 2,701 edges, was visualized using Cytoscape v3.8 software. As analysis results of the MCODE plug-in showed, 39 subnetworks were identified, and the first cluster (Figure 5A) was related to mitotic cell cycle, PID Aurora B pathway, regulation of the cell cycle process and chromosome segregation, PID FOXM1 pathway, and PID PLK1 pathway (Figure 5B). Hub genes such as topoisomerase II alpha (TOP2A), cyclin-B2 (CCNB2), KIF11, cyclin-A2 (CCNA2), cell division cycle 20 (CDC20), cell division cycle–associated protein 8 (CDCA8), KIF20A, benzimidazoles 1 homolog beta (BUB1B), targeting protein for Xenopus kinesin-like protein 2 (TPX2), and KIF2C were identified using an MCC algorithm (Figure 5C). Enrichment analysis indicated that the hub genes were significantly enriched in cell division, nuclear division, mitotic cell cycle, microtubule cytoskeleton, PID Aurora B pathway, PID PLK1 pathway, and PID FOXM1 pathway (Figure 5D).

In addition, PPI networks and relevant hub genes of 31 upregulated metabolic DEGs (Figures 5E,F) and 30 downregulated metabolic DEGs (Figures 5G,H) were both constructed and identified. Further, the KM plotters of significant DEGs and hub genes indicated that the expression levels of GHRL, centrosomal protein 55 (CEP55), and POLR1A were associated with survival outcomes in patients with GC (Figure 5I).

### Mutated Genes Analysis Based on The Cancer Genome Atlas Database

**FIGURE 6:**
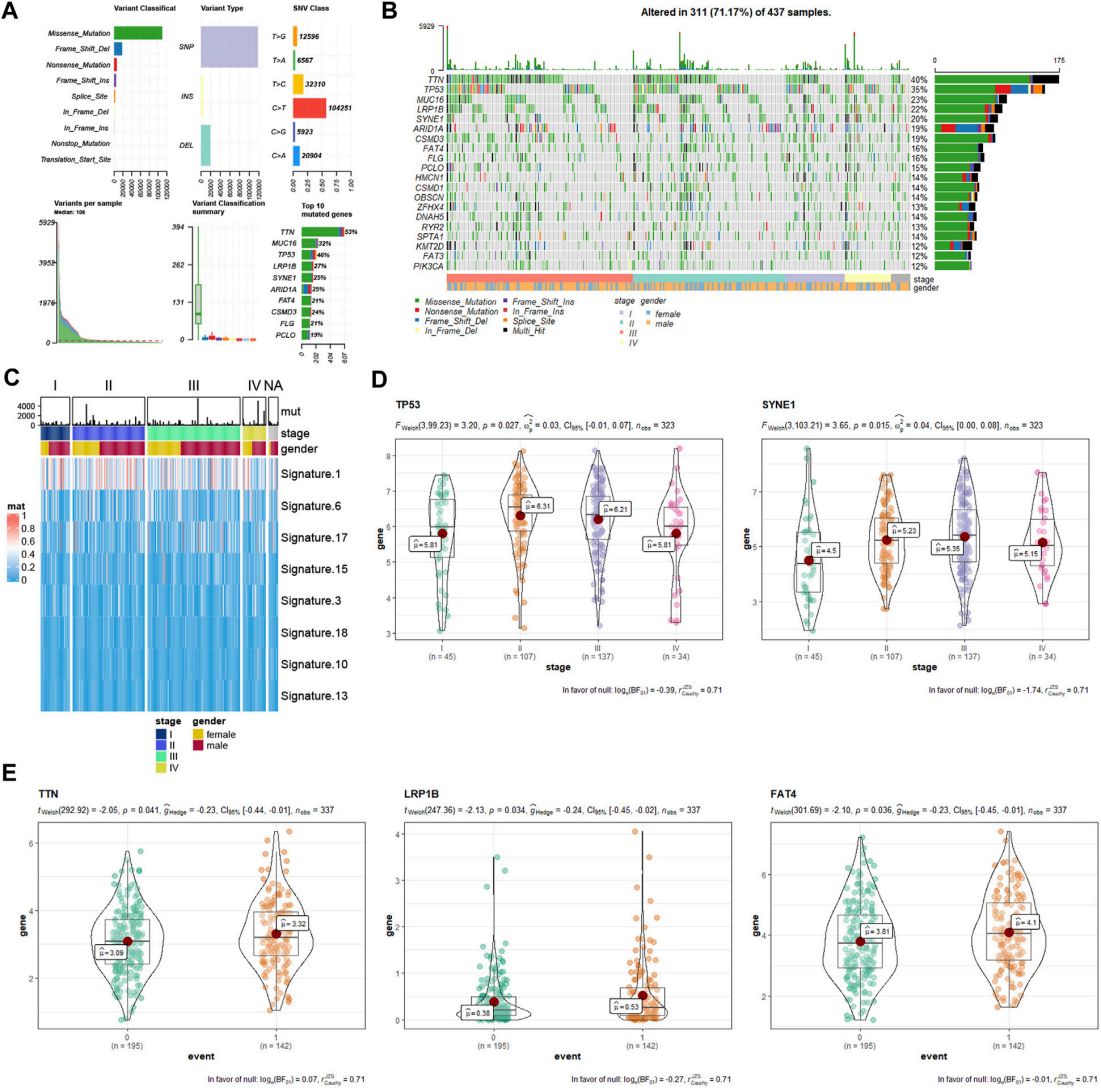
Gene mutation information of GC samples from TCGA database. **(A)** mutation profile landscape in GC samples. **(B)** waterfall plot showing the mutation details of every gene in every sample. **(C)** heatmap showing the significant signatures of mutated genes. **(D)** correlation analysis between tumor stage with mutated genes (TP53 and SYNE1). **(E)** correlation analysis between survival event (0, alive; 1, death) with mutated genes (TTN, LRP1B, and FAT4). GC, gastric cancer; TCGA, The Cancer Genome Atlas.

A summary of gene mutation information of GC patients from TCGA database is shown in Figure 6A. Somatic mutation profiles for 437 GC patients were retrieved. And we used the waterfall plot to present the mutation data for every gene in every sample (Figure 6B). The significant signatures of mutated genes were also explored (Figure 6C). Further, we assessed the effects of the top 10 mutated genes (TTN, TP53, MUC16, LRP1B, SYNE1, ARID1A, CSMD3, FAT4, FLG, and PCLO) in tumor progression. As the statistical analysis showed, TP53 and SYNE1 were associated with tumor stage (Figure 6D), while TTN, LRP1B, and FAT4 were associated with survival events (Figure 6E).

## Discussion

As one of the most common malignant tumors with high mortality, GC has always been a serious disease threatening human health. Risk prediction, early diagnosis, and precise therapies are considered the essential measures to improve the prognosis of GC patients. Owing to complex BP in GC occurrence and development, however, existing markers and therapeutic targets could not significantly improve diagnosis or 5-year survival rate (Ajani et al., 2016; Wang et al., 2019). Therefore, a study on the pivotal mechanism of GC occurrence and development is urgently needed, which helps select sensitive and specific biomarkers or therapeutic targets.

In this study, the gene expression data of GSE19826, GSE26899, GSE33335, GSE63089, GSE79973, and GSE118916 were integrated through a meta-analysis method. Then enrichment analysis, PPI network construction, and hub gene identification were performed based on integrated data. In total, 1,065 upregulated and 164 downregulated DEGs throughout GC development were screened out. Enrichment analyses of GO terms and KEGG pathways indicated that DEGs could lead to the incidence and exacerbation of GC by affecting DNA replication, cell and chromosome division, and related metabolic pathways. Furthermore, ECM–receptor interaction and PI3K-Akt were also KEGG pathways that DEGs significantly enriched. The tumor microenvironment (TME), a complex ecosystem composed of ECM, peripheral blood vessels, other non-malignant cells, and signaling molecules, has been proved to be important for tumor invasion, progression, and chemoresistance (Ran et al., 2021). The biological function of PI3K-Akt signaling in GC progression has been well established, which could regulate tumor cell growth, proliferation, apoptosis, and energy metabolism. An activated PI3K-Akt signaling pathway could promote GC progression by enhancing glycolysis, stabilizing mitochondrial membrane potentials, and inhibiting tumor cell apoptosis (Xu et al., 2020). In addition, abundant metabolic pathways were found to be associated with GC, revealing the close connection between the metabolic system and the identified DEGs (Tian et al., 2020). To date, accumulated evidence has suggested that cancer is a metabolic disease, in which cells have lost their normal checks on cell proliferation, resulting in excessive bioenergetic and biosynthetic needs. Therefore, cancer cells must alter their metabolism to sustain such a high demand (Kroemer and Pouyssegur, 2008; Li et al., 2013; Duda et al., 2020). Large amounts of purines and pyrimidines are required for cell proliferation, DNA replication, and energy supply of GC tissues; thus, pyrimidine metabolism and purine metabolism are metabolic pathways upregulated DEGs significantly enriched. Moreover, the majority of downregulated DEGs converged on metabolic pathways such as metabolism of retinol, drug–cytochrome P450, glycolysis/gluconeogenesis, histidine, alanine, aspartate, glutamate, and tyrosine. It has long been recognized that tumor metabolism preferentially relies on glycolysis instead of oxidative phosphorylation of glucose despite the status of oxygen supply, and this type of metabolism is known as the Warburg effect (Kroemer and Pouyssegur, 2008). As a result, key enzymes in the oxidative phosphorylation of glucose are downregulated for cancer cell proliferation and tumorigenicity. Fructose-1,6-bisphosphatase-2 (FBP2), one kind of enzyme participating in glycogen synthesis from carbohydrate precursors, can catalyze the hydrolysis of fructose-1,6-bisphosphate to fructose-6-phosphate and inorganic phosphate in glucose metabolism. FBP2 underexpression may contribute to GC tumor development by stimulating glucose metabolism and inhibiting cell proliferation (Li et al., 2013; Duda et al., 2020). In recent years, glucolipid metabolism therapy of tumors has become a research hotspot, which aims to inhibit the proliferation and metastasis of tumor cells by controlling glycolysis and increasing fat for energy (Hur et al., 2013; Sun et al., 2018). Gastric acid secretion, another important pathway that downregulated DEGs enriched, has been reported by few studies. GC was considered to be associated with gastritis, and intestinal metaplasia developing in atrophic gastritis was believed to be a step in the gastric carcinogenic process. Therefore, patients with GC had reduced gastric acid secretion because of oxyntic atrophic gastritis (Waldum and Rehfeld, 2019; Liu et al., 2020a). Furthermore, decreased GHRL in blood also contributed to this phenomenon.

Up to now, tumor markers such as CEA, CA125, CA199, and CA72-4 have been used as indicators for GC diagnosis. Her2, epidermal growth factor receptor (EGFR), TP53, and PI3K are key oncogenes reported to work (Li et al., 2022; Zhao et al., 2022). Besides these, Xie et al. (2015) established a multi-index prediction model based on the six kinds of biomarkers (CEA, CA199, H.P., P53, PG Ⅰ, and PG Ⅱ), which was designed to achieve early screening and therapeutic evaluation of GC patients. However, the above biomarkers and therapeutic targets had insufficient specificity in the early diagnosis of GC and could not accurately evaluate tumor progression and survival time (Gao and Yang, 2022). In this study, COL1A2, TIMP1, THY1, and BGN were identified as significant DEGs for both the GC with ES and the GC with LS. Further analysis showed that the expression levels of the above four DEGs were associated with tumor stage, and the expression level of GHRL was associated with LNR and survival outcome. Recurrence and metastasis, the primary factors affecting the survival outcome of GC patients, are also the hotspots of current research. The above DEGs may influence tumor stage and LNR by affecting GC recurrence and metastasis. COL1A2 gene can affect cell proliferation, differentiation, adhesion, and metastasis by encoding type I collagen—the most widely expressed collagen among the fibrous collagen family (Pan et al., 2021; Xu et al., 2021). TIMP1 is a natural collagenase inhibitor that can inhibit apoptosis, induce angiogenesis, and stimulate cell proliferation, which may be directly involved in the progression and metastasis of cancers such as GC, breast cancer, and colon cancer (Zhang et al., 2020; Liu et al., 2021). THY1, also known as the CD90 gene, plays an important regulatory role in the cellular interactions between a cell and its matrix. Moreover, previous studies indicated that the THY1 gene can inhibit GC cells’ apoptosis by regulating secreted protein acidic and rich in cysteine protein’s expression levels (Wu et al., 2019; Wang et al., 2021). As an important component of ECM proteins, BGN seems to play an important role in the oncogenesis and progression of various cancers. According to the research by Hu et al. (2016), BGN may promote cancer progression through the chronic activation of tumor angiogenesis, so high expression of BGN was observed in advanced GC. In addition, tight connections between the above four hub genes could be found; thus, definitive linkages and interaction mechanisms needed to be further explored. Therefore, COL1A2, TIMP1, THY1, BGN, and GHRL have great potential in applications such as early screening of GC patients, prediction of therapeutic outcomes, and real-time dynamic monitoring the progress of GC in near future.

TOP2A, CCNB2, KIF11, CCNA2, CDC20, CDCA8, KIF20A, BUB1B, TPX2, and KIF2C were identified as the top 10 hub genes among all DEGs. TOP2A is one kind of type II DNA topoisomerases that can relax negative and positive supercoiling during replication and transcription. The expression level of the TOP2A gene can reflect tumor proliferation and was reported to be associated with peritoneal and hematogenous recurrence. Thus, TOP2A may be utilized as a specific drug target for malignant tumors such as GC (Hou et al., 2020). As previous studies found, CCNB2 was a cell cycle–related gene that can promote the proliferation and tumor growth of GC cells (Liu et al., 2020b). Similar to CCNB2, CCNA2 is a highly conserved cyclin that plays a critical role in the control of the cell cycle at G1/S and in the G2/M transition (Lee et al., 2020). Kinesin superfamily (KIF), a group of proteins that possess ATPase activity and motion characteristics, participate in numerous cellular biological activities such as mitosis and meiosis. Kruppel (KIF) 11, KIF20A, and KIF2C genes have been illustrated as genes that might function as oncogenes in GC (Imai et al., 2017; Sheng et al., 2018). Several lines of evidence have shown that CDC20 plays a vital role in the correct functioning of the spindle assembly checkpoint (SAC), and overexpression of the CDC20 gene is related to intestinal histology and favorable clinicopathologic parameters in GC (Kim et al., 2019). In addition, multidomain protein kinase budding uninhibited by BUB1B may contribute to the process of SAC, which can delay the separation of sister chromatids to prevent defects in segregation (Hudler et al., 2016). CDCA8, also called Borealin/Dasra B, is a crucial cell cycle–regulated chromosomal passenger protein, and its nuclear accumulation is correlated with a poor prognosis for GC (Chang et al., 2006). TPX2 is a microtubule-associated protein that relates to chromosomal instability and helps format normal bipolar spindles and chromosome segregation (Tomii et al., 2017).

Further, the expression levels of GHRL, CEP55, and POLR1A were proved to be associated with survival outcomes in patients with GC. GHRL, a small peptide characterized as the ligand of the growth hormone secretagogue receptor (GHSR), plays role in stimulating pituitary growth hormone release, the regulation of energy balance, gastric acid release, appetite, insulin secretion, gastric motility, and the turnover of the gastric and intestinal mucosa. Previous studies have shown that GHRL works in several key processes of cancer progression, such as cell proliferation, migration, and invasion. The action mechanism of GHRL in promoting or inhibiting cancer progression, however, is still unclear. The regulation of the GHRL–GHSR axis may play a potential critical role (Lin and Hsiao, 2017). As a member of the centrosomal relative protein family, CEP55 has been reported to participate in cell cycle regulation. Tao’s study showed that ectopic overexpression of CEP55 could enhance the cell proliferation, colony formation, and tumorigenicity of GC cells, and CEP55 knockdown induces cell cycle arrest at the G2/M phase in GC cells. Besides, the expression of CEP55 can affect the PI3K/AKT/p21 signaling pathway and cyclin pathway-related proteins (Tao et al., 2014). Folate receptor gene family has a high affinity for folic acid and several reduced folic acid derivatives, and it mediates delivery of 5-methyltetrahydrofolate to the interior of cells. Moreover, mature FOLR1 is an N-glycosylated protein that is predominantly expressed on epithelial cells and is dramatically upregulated on many carcinomas (Kim et al., 2018).

## Conclusion

In this study, six eligible microarray datasets were integrated to present the gene expression signatures of GC relative to normal gastric tissues using ImaGEO meta-analysis. COL1A2, TIMP1, THY1, and BGN were identified and verified as significant DEGs throughout GC progression, and the above DEGs are expected to be used as the target molecules in GC diagnosis and therapy. In the future, basal experimentation and clinical tests are needed to verify their roles in early screening and tumor preventing and controlling of GC patients. As enrichment analysis showed, the upregulated DEGs mainly enriched in DNA replication, cell cycle, and ECM–receptor interaction, while most of the downregulated DEGs were related to metabolism. TOP2A, CCNB2, KIF11, CCNA2, CDC20, CDCA8, KIF20A, BUB1B, TPX2, and KIF2C were identified as the top 10 hub genes among all DEGs. Further, the expression levels of GHRL, CEP55, and POLR1A were proved to be associated with survival outcomes in patients with GC. The interaction between hub genes, the intervention mechanism of hub genes on tumors, and the association between hub genes with survival outcomes are the main direction for the next research step.

The identified DEGs, hub genes, and signaling pathways may help us understand the molecular mechanisms of gastric tumor and discover new biomarkers and therapeutic targets for gastric tumor.

## Data Availability

The original contributions presented in the study are included in the article/Supplementary Material, and further inquiries can be directed to the corresponding author.
